# Functional ovarian reserve in women with Hodgkin and non-Hodgkin lymphomas

**DOI:** 10.3389/fonc.2026.1965843

**Published:** 2026-10-09

**Authors:** Francesca Filippi, Nicole Serra, Sabrina Comana, Giorgia Di Stefano, Marco Reschini, Paola Vigano’, Edgardo Somigliana

**Affiliations:** 1 Infertility Unit, Fondazione IRCCS Ca’ Granda Ospedale Maggiore Policlinico, Milan, Italy; 2 Department of Clinical Sciences and Community Health, Università degli Studi, Milan, Italy

**Keywords:** cancer, fertility preservation, lymphoma, oocytes, ovarian reserve

## Abstract

**Introduction:**

Hematological malignancies may impair ovarian function even before chemotherapy begins, raising concerns about the effectiveness of oocyte cryopreservation. However, available evidence remains conflicting, partly because of confounding factors and suboptimal study designs. This study investigated whether women with lymphoma show reduced ovarian reserve or impaired ovarian response compared with women without cancer and women with non-hematologic malignancies.

**Methods:**

We conducted a retrospective analysis of women with Hodgkin and non-Hodgkin lymphomas who underwent oocyte cryopreservation for fertility preservation at our Institution. Each patient was age- and study period-matched to two control groups: one comprising women without cancer undergoing Assisted Reproductive Technologies for infertility, allowing comparison of ovarian reserve markers (Anti-Müllerian Hormone, AMH, and antral follicle count, AFC), and another comprising women with non-hematologic solid tumors to compare ovarian stimulation outcomes.

**Results:**

The first comparison included 66 cases and 66 controls without cancer. Median [interquartile range – IQR] serum AMH was 1.97 [1.14-3.23] and 2.94 [2.12-4.60], respectively (p=0.002). However, AFC was similar (18 [12-24] versus 15 [11-21], p=0.16). The second comparison involved 74 cases and 74 non-hematologic cancer controls. Ovarian response was similar between groups, with a median [IQR] number of frozen mature oocytes of 10 [6–15] and 13 [6–16], respectively (p=0.56).

**Conclusion:**

Early alterations in follicular function may occur in women with lymphoma. Nevertheless, ovarian response to stimulation appears preserved, supporting the effectiveness of fertility preservation.

## Introduction

In the last decade, treatments for onco-hematological cancers have improved, markedly increasing the overall survival rates. Chemotherapy and radiotherapy represent the main treatments for these cancers but can impair ovarian function (1, 2). Reproductive and oncological international guidelines uniformly recommend discussing fertility preservation with reproductive-aged women prior to embarking on these treatments (3, 4). Hematological malignancies are the second most common indication for fertility preservation in women of reproductive age, with oocyte cryopreservation being the preferred approach (3, 4).

Several studies have demonstrated a diminished functional ovarian reserve in patients diagnosed with Hodgkin and non-Hodgkin lymphomas even before initiation of chemotherapy (5, 6). Moreover, some evidence favors the idea that ovarian response to stimulation in women diagnosed with hematological cancers may be lower compared to healthy controls or to women with other solid malignancies (7–9). A recent systemic review highlighted a detrimental effect of hematological malignancies on ovarian function when comparing Anti-Mullerian Hormone (AMH) serum levels of affected women with healthy controls (10).

However, data on whether oncological diseases influence the number or quality of oocytes retrieved after ovarian stimulation remain inconsistent. Such discrepancies may be explained by confounding factors and limitations in study designs. This topic is clinically relevant, as it may inform patient counselling, and scientifically intriguing, since evidence that a systemic oncological disease can affect ovarian physiology could open new areas of research.

The aim of our study was to rigorously assess the impact of hematological cancers on functional ovarian reserve. To this purpose, information obtained from affected cases undergoing egg freezing were compared to both women without malignancies and to women with other solid cancers.

## Materials and methods

Women who were referred to the Infertility Unit of Policlinico Hospital, Milan, between January 2013 and April 2024 were retrospectively reviewed. Patients undergoing oocyte cryopreservation for hematologic malignancies were initially selected as the study group. Inclusion criteria were as follows: (i) age between 18 and 39 years; (ii) both ovaries present and easily visualized by transvaginal ultrasound scan; (iii) starting of ovarian stimulation with gonadotropins. Exclusion criteria were as follows: (i) diagnosis of gynecological pathologies involving the ovaries; (ii) previous ovarian surgery; (iii) ultrasound or surgical diagnosis of endometriosis; (iv) polycystic ovary syndrome; (v) previous gonadotoxic treatments; (vi) incomplete charts.

Two control groups were subsequently identified, for which the same inclusion and exclusion criteria were applied. The first consisted of age-matched women without malignancies (non-oncologic controls) who underwent ART for male factor infertility, tubal factor infertility, or preimplantation genetic testing for monogenic disorders (PGT-M). This control group is expected to reflect the general population, and the comparison focused on ovarian reserve biomarkers. Women assuming hormonal contraception were excluded from both cases and controls due to its potential impact on ovarian reserve variables. Response to ovarian stimulation was not compared between the two groups because deemed unreliable and potentially biased (different protocols and gonadotropin dosages).

The second control group included age-matched women diagnosed with other solid malignancies who underwent oocyte cryopreservation for fertility preservation. This comparison focused on ovarian stimulation outcomes. For both control groups, matching was performed by age (± 1 year) and study period, selecting the closest eligible woman within the same temporal window.

The study was approved by the local Ethics Committee. In our unit, all patients undergoing oocytes cryopreservation and ART signed a written consent for their data to be used for research purposes.

Given the emergency setting of fertility preservation procedures, all women with cancer scheduled for oocyte freezing were treated with a random start protocol. Ovarian stimulation was performed using a GnRH antagonist protocol until December 2019. Since January 2020 a progestin primed protocol was systematically introduced, as described previously (11). In case of hormone sensitive tumors, letrozole 5 mg was administered orally once daily during the stimulation period. If the patient was taking contraceptive pill, its use was interrupted with the beginning of the stimulation.

Tested biomarkers of functional ovarian reserve included Antral Follicle Count (AFC) and AMH. For the outcome of ovarian stimulation, the main variables studied were the number of mature and frozen oocytes. Clinical information regarding patients’ anamnesis, oncological diagnoses, ovarian reserve biomarkers and ovarian stimulation outcomes was obtained from patients’ charts. AMH assessments were performed in our Centre. The commercially available Beckman Coulter Gen II ELISA assay on the automated GEMINI platform (STRATEC Biomedical AG, Germany) after dilution with assay buffer was used (12). To assess AFC, all identifiable antral follicles 2–10 mm in diameter were recorded, as previously described (13). Scans for the assessment of AFC were performed by six gynaecologists with long-lasting expertise in ultrasound and infertility. Low AMH was defined as a serum value < 0.7 ng/ml and low AFC as a total count ≤ 7 (14). Low response was defined as the retrieval of ≤ 3 mature oocytes (15).

The sample size calculation was performed focusing on the proportion of women with low ovarian reserve or low response. Our local data indicated that this occurs in about 10% of women. Setting type I and II errors at 0.05 and 0.20, a sample size of at least 60 women per group would consent to demonstrate a 4-folds increase in the risk. Data collected were transferred in Statistical Package for Social Science (SPSS 26.0, IL, USA) database for subsequent analyses. Differences were evaluated using Chi square test, the Student *t* test and the Mann-Whitney test, as appropriate. Data are reported as number (percentage), mean ± Standard Deviation (SD) or median [Interquartile range-IQR]. For the main continuous outcomes, Hodges–Lehmann estimates of paired differences with 95% confidence intervals were calculated. Differences were defined as values in women with hematological cancers minus those in their matched controls. A p value <0.05 was considered statistically significant.

## Results

Overall, seventy-four women diagnosed with hematological malignancies fulfilled our initial selection criteria. Sixty-six (89%) had Hodgkin lymphoma, and eight had non-Hodgkin lymphoma. For the first comparison, we excluded eight cases because they were assuming estroprogestins at the time of recruitment, leaving 66 women for data analyses. They were matched to 66 non-oncologic controls. This latter group included 52 women (78%) who had male factor infertility, 11 (17%) with a tubal factor, and 3 (5%) who underwent preimplantation genetic testing. Baseline characteristics and ovarian reserve biomarkers were compared. Results are shown in **Table 1**. Serum AMH was significantly lower in onco-hematologic patients (p=0.002). A trend toward a higher frequency of women with low AMH also emerged. In contrast, AFC did not differ. The Hodges–Lehmann estimated paired differences, calculated as hematological minus matched control values, were −0.95 ng/mL (95% CI, −1.58 to −0.33) for AMH and +2.0 follicles (95% CI, −0.5 to +4.5) for AFC.

**Table 1:** Comparison between women with lymphomas and age matched controls without malignancies scheduled for ART.

Characteristics	Lymphomas	Non-cancer women	p
	n=66	n=66	
Age (years)	30 [26 - 33]	30 [26 - 34]	0.99
BMI (Kg/m^2^)	21.0 [19.4 - 22.8]	22.1 [20.4 - 24.2]	0.07
Previous pregnancies	10 (15%)	16 (24%)	0.27
Regular menstrual cycles ^a^	59 (91%)	62 (94%)	0.53
AMH (ng/ml)	1.97 [1.14 - 3.23]	2.94 [2.12 - 4.60]	0.002
AMH <0.7 ng/ml	6 (9%)	1 (2%)	0.05
AFC	18 [12 - 24]	15 [11 - 21]	0.16
AFC ≤ 7	4 (6%)	5 (8%)	0.73

Data are reported as median [interquartile range] or number (percentage).

Women taking estroprogestins at the time of recruitment were excluded.

^a^The information is missing for one woman with lymphoma.

BMI, Body Mass Index; AMH, Anti-Müllerian hormone; AFC, Antral follicle count.

For the second comparison, all 74 women with lymphomas were included. They were matched to 74 women with other cancers. Diagnoses of the latter included 41 women with breast cancer (55%), 11 with melanoma (15%) and the remaining 22 (30%) other solid malignancies such as gastrointestinal, central nervous system malignancies or peritoneal mesothelioma. Baseline characteristics and ovarian reserve biomarkers were compared. Results are shown in **Table 2**. Again, serum AMH resulted lower in women with lymphomas (p=0.01), even if the proportion of subjects with low AMH was similar and AFC did not differ. The paired difference (95% CI) of AMH and AFC was -0.86 (-1.42 to -0.30) ng/ml and -2.5 (-5.5 to +0.5), respectively. Comparison of the ovarian stimulation variables between the two cancer groups are shown in **Table 3**. Except for the expected use of letrozole in some women belonging to the group of other solid tumors, no significant differences were outlined as for the duration of stimulation, total doses of gonadotropins, number of cancelled cycles for inadequate response, number of follicles > 11 mm on the day of trigger, number of retrieved oocytes and number of cryopreserved (mature) oocytes. The paired difference (95% CI) in the number of retrieved and cryopreserved oocytes was 0.0 (-3.0 to +2.5) and 0.0 (-2.5 to +2.0), respectively. The Spearman correlation indexes between the number of oocytes retrieved and AMH and AFC were 0.62 and 0.54, respectively (p<0.001 for both). For the number of cryopreserved oocytes, the Spearman correlation indexes were 0.60 and 0.52, respectively (p<0.001 for both).

**Table 2:** Comparison between women with lymphomas and those with other cancers: baseline characteristics and ovarian reserve biomarkers.

Characteristics	Lymphomas	Other cancers	p
	n=74	n=74	
Age (years)	30 [26 - 33]	30 [26 - 33]	0.99
BMI (Kg/m^2^)	21.0 [19.4 - 22.8]	21.7 [19.7 - 23.4]	0.13
Previous pregnancies	12 (16%)	11 (15%)	1.00
Regular menstrual cycles ^a^	64 (89%)	65 (90%)	1.00
Current use of estroprogestins	8 (11%)	15 (20%)	0.17
Pregnancy seeking	5 (7%)	9 (12%)	0.40
AMH (ng/ml)	1.97 [1.14 - 3.23]	3.11 [1.54 - 4.52]	0.01
AMH <0.7 ng/ml	6 (8%)	4 (5%)	0.51
AFC	18 [12 - 24]	20 [13 - 26]	0.18
AFC ≤ 7	4 (5%)	3 (4%)	0.70

Data are reported as median [interquartile range] or number (percentage).

^a^The information is missing for two women in both groups.

BMI, Body Mass Index; AMH, Anti-Müllerian hormone; AFC, Antral follicle count.

**Table 3:** Comparison between women with lymphomas and those with other cancers: ovarian stimulation outcome.

Characteristics	Lymphomas	Other cancers	p
	n=74	n=74	
Protocol of stimulation			0.41
GnRH antagonist	34 (46%)	40 (54%)	
Progestin-Primed Ovarian Stimulation	40 (54%)	34 (46%)	
Use of letrozole	0 (0%)	19 (26%)	< 0.001
Total UI of gonadotropins	2,300 [1,800 - 2,600]	2,150 [1,800 - 2,600]	0.71
Duration of stimulation	11 [10 - 12]	11 [10 - 12]	0.57
Cancelled cycle (insufficient response)	2 (3%)	1 (1%)	1.00
Total number of follicles ^a^	17 [12 - 21]	18 [12 - 22]	0.62
Total number of retrieved oocytes ^a^	12 [7 - 18]	15 [7 - 20]	0.64
Number of retrieved oocytes ≤ 3 ^a^	6 (8%)	7 (9%)	0.77
Total number of cryopreserved oocytes ^a^	10 [6 - 15]	13 [6 - 16]	0.56
Number of cryopreserved oocytes ≤ 3 ^a^	8 (11%)	10 (14%)	0.62

Data are reported as median [interquartile range] or number (percentage).

GnRH, Gonadotropin Releasing Hormone; IU, International Unit; AMH, Anti-Müllerian hormone; AFC, Antral follicle count.

^a^ Refers to cases that were not cancelled (72 and 73, respectively).

## Discussion

In women with lymphomas, the two biomarkers of ovarian reserve tested in this study, i.e., AFC and AMH, had different trends. The latter resulted lower compared to both non-oncologic controls and subjects diagnosed with other solid malignancies while the former did not differ. In addition, ovarian response to stimulation was not affected compared to women with other cancers. Clinically, this study is reassuring because women with lymphomas are not expected to have a less effective program of oocyte freezing.

Oocyte vitrification represents the standard of care for fertility preservation in post-pubertal women diagnosed with malignancies. The ovarian response to stimulation is a key determinant of the success of this procedure. However, scientific evidence remains conflicting regarding whether the underlying type of cancer influences ovarian response, particularly in patients with onco-hematological malignancies. A recent systematic review and meta-analysis (10) including eight observational studies, showed lower serum AMH levels in patients with hematologic cancer when compared to healthy controls. The mean difference (MD) was -1.0 ng/ml (95%CI: -1.8 to -0.3) despite a younger age of the former group (MD -4.2, 95% CI -6.2 to -2.1; p<0.0001) and a similar number of retrieved oocytes (MD 0.20, 95% CI -0.80 to +1.20; p=0.69). Our findings of lower levels of serum AMH but similar AFC and number of retrieved oocytes are therefore consistent with previous evidence. The main strength of our study is the scrupulous attention given to confounders. Main precautions included matching for age and study period, a scrupulous assessment of the studied variables (all tests for AMH were performed in our unit and only few gynaecologists performed all the US scans) and the selection of two different control groups according to the investigated outcome. To the best of our knowledge, no previous studies compared ovarian reserve variables in hematologic malignancies with both age-matched women without cancer and with age-matched women with other solid tumors.

Decreased levels of AMH and AFC have been previously associated with the use of estroprogestin therapy (16) when compared with non-users. Our control group of women without cancer were selected among those undergoing ART (allowing recruitment in the same study period of subjects with complete information on ovarian reserve), and none of them were taking oral contraceptives. For this reason, we excluded women with lymphomas undergoing estroprogestin therapy for this first comparison. Interestingly, in the second comparison aimed at studying cycle outcome and including estroprogestins users, a slightly higher proportion of users was present in the group of patients with other solid malignancies, possibly attenuating the observed differences in AMH values between groups.

Although Danis et al. (17) showed similar fertility preservation results in conventional and random-start approaches, to avoid any bias due to different types of stimulation protocols, we compared ovarian stimulation results only among cancer patients, when random-start protocol was systematically applied. The use of letrozole in some women in the control group represents a possible confounder. However, this drug was not previously shown to influence the number of retrieved and vitrified mature oocytes (18).

The isolated finding of a lower AMH is intriguing and difficult to explain. It may be speculated that the presence of a systemic malignancy such as lymphomas may indirectly interfere with the mechanisms of the recruitment of primordial follicles. AMH is primarily secreted by secondary and early antral follicles, which cannot be visualized as antral follicles on ultrasound (16). As a marker of functional ovarian reserve, AMH reflects earlier stages of follicular development compared to AFC. Therefore, disturbances in the initial stages of follicular recruitment may lead to a decline in AMH levels before any detectable reduction in AFC occurs. It cannot be excluded that, if lymphomas were to remain untreated for additional weeks or months, both AFC and ovarian response might eventually decrease as well. To indirectly test this hypothesis, it would be necessary to determine the time elapsed since disease onset and to assess whether a biological gradient exists. Unfortunately, such information is not reliably obtainable, and our hypothesis remains speculative.

Noteworthy, hematologic cancers are associated with a systemic inflammatory response, characterized by increased endothelial growth factor, transforming growth factor (TGF)-β, cytokines (interleukin-6), and C-reactive protein (19, 20). These pro-inflammatory mediators have been implicated in disrupting normal ovarian physiology. In fact, animal studies have shown that the increased expression of TGF-β can inhibit folliculogenesis, oocyte maturation, and ovulation (21, 22). Such alterations may impair granulosa cell function, which is critical for AMH production, thereby contributing to reduced circulating AMH levels (23). Furthermore, histological analyses of fresh ovarian cortex tissue from patients with Hodgkin lymphoma have revealed a significantly increased number of vacuolated follicles compared with healthy controls, suggesting a damage to germ-cells prior to cancer treatment for hematologic malignancies (24). Finally, metabolic disturbances, such as the higher catabolic status associated with lymphomas, may contribute to hypothalamic-pituitary-ovarian axis dysregulation. Increased stress hormone levels such as prolactin, and endogenous opiate production, suppress gonadotropin levels, thus further compromising ovarian function.

The single center and retrospective design represent limitations of our study. Moreover, data was mostly from Hodgkin lymphoma patients, thus not allowing any robust sub-analysis for non-Hodgkin lymphomas. For the same reason, we could not reliably explore the impact of specific lymphomas’ characteristics on the risk of low AMH. Noteworthy, Goldenberg et al. (9) outlined an independent effect of clinical cancer stage, the presence of adverse clinical symptoms or biological parameters, but we could not replicate this analysis. There is the need for future large studies that investigate whether the reduction in AMH is associated with some characteristics of the malignancy (such as disease stage, symptoms, biological biomarkers, cell lineage, cellular morphology, immunophenotype, and molecular/genetic mutations).

Collectively, these findings support the hypothesis that lymphomas can adversely affect functional ovarian reserve even before the onset of gonadotoxic therapy. However, the long-lasting folliculogenesis process in humans tempers this effect at the time of diagnosis. This detrimental impact is masked when considering AFC and follicular development because follicles contributing to AFC and oocytes yield have initiated their developmental journey several months before (25), when the systemic derangement associated to the malignancy was yet unremarkable. There is no evidence to suggest whether this disruption of the early steps of folliculogenesis is transient (i.e., without long-term effect) or permanent. Affected women must be treated and once recovered, it is not possible to distinguish chemotherapy-associated damage from a direct injury caused by the malignancy itself. Noteworthy, only few studies investigated long term fertility issues in women treated for hematological malignancies. Interestingly, a recent contribution suggested that biomarkers of ovarian reserve may partly recover in the long term (26). Moreover, it must be emphasized that there is strong evidence questioning the relevance of low ovarian reserve for natural conception (27). In the general population, ovarian reserve may not reflect natural fertility, provided that the remnant pool of primordial follicles is sufficient to ensure regular ovulatory cycles There is the need to collect evidence on this aspect also for the specific group of women with injured ovarian reserve because of hematological malignancies.

On the other hand, our study is reassuring for short term clinical purposes. Ovarian response to stimulation appears to be largely unaffected in women with lymphomas, who in most cases can cryopreserve an adequate number of mature oocytes. Moreover, for counselling purposes, AFC may represent a more appropriate marker than AMH in this clinical setting. Interestingly, the correlation between AMH and oocytes yield remains also in women with lymphomas suggesting that AMH could also be used in this population but must be interpreted more cautiously. It can indeed underestimate the response. Finally, it must be emphasized that, additional studies are required to generate evidence on the developmental competence and quality of the vitrified oocytes. It should be noted that the number of mature oocytes retrieved does not directly translate into fertilization potential, embryo quality, implantation success, or pregnancy rates. Hence, future research should aim to elucidate the broader impact of hematologic malignancies on the use and ultimate effectiveness of fertility preservation.

## Data Availability

The raw data supporting the conclusions of this article will be made available by the authors, without undue reservation.
